# Comparison of outcomes on hypoxia-inducible factor prolyl hydroxylase inhibitors (HIF-PHIs) in anaemia associated with chronic kidney disease: network meta-analyses in dialysis and non-dialysis dependent populations

**DOI:** 10.1093/ckj/sfad298

**Published:** 2023-12-07

**Authors:** Alfred Sackeyfio, Renato D Lopes, Csaba P Kovesdy, Aleix Cases, Steve A Mallett, Nick Ballew, Tom J Keeley, Viviana Garcia-Horton, Rajeev Ayyagari, Rodrigo Refoios Camejo, Kirsten L Johansen, Alexander J Sutton, Indranil Dasgupta

**Affiliations:** GSK, Stevenage, Hertfordshire, UK; Duke University Medical Center, Duke Clinical Research Institute, Durham, NC, USA; University of Tennessee Health Science Center, Memphis, TN, USA; Universitat de Barcelona, Barcelona, Spain; GSK, Brentford, London, UK; GSK, Collegeville, PA, USA; GSK, Brentford, London, UK; Analysis Group Inc., NY, USA; Analysis Group Inc., Boston, MA, USA; GSK, Brentford, London, UK; Hennepin Healthcare, University of Minnesota, Minneapolis, MN, USA; Department of Population Health Sciences, University of Leicester, Leicester, UK; Department of Renal Medicine, University Hospitals of Birmingham NHS Foundation Trust, Birmingham, UK; Warwick Medical School, University of Warwick, West Midlands, UK

**Keywords:** anaemia, chronic kidney disease, hypoxia-inducible factor prolyl hydroxylase inhibitors, network meta-analysis, outcomes

## Abstract

**Background:**

Hypoxia-inducible factor prolyl hydroxylase inhibitors (HIF-PHIs) are oral alternatives to current standard-of-care treatments for anaemia in chronic kidney disease (CKD). We conducted network meta-analyses to indirectly compare clinical outcomes for three HIF-PHIs in dialysis and non-dialysis populations with anaemia in CKD.

**Methods:**

The evidence base comprised phase III, randomised, controlled trials evaluating daprodustat, roxadustat, or vadadustat. Three outcomes were evaluated: efficacy [change from baseline in haemoglobin (Hgb)], cardiovascular safety [time to first major adverse cardiovascular event (MACE)] and quality of life [change from baseline in 36-Item Short Form Health Survey (SF-36) Vitality score]. Analyses were performed separately for all patients and for erythropoiesis-stimulating agent (ESA) non-users at baseline (non-dialysis population) or prevalent dialysis patients (dialysis population). Bayesian Markov Chain Monte Carlo methods with non-informative priors were used to estimate the posterior probability distribution and generate pairwise treatment comparisons. Point estimates (medians of posterior distributions) and 95% credible intervals (CrI) were calculated.

**Results:**

Seventeen trials were included. In non-dialysis patients, there were no clinically meaningful differences between the three HIF-PHIs with respect to Hgb change from baseline [all patients analysis (total *n* = 7907): daprodustat vs. roxadustat, 0.09 g/dL (95% CrI −0.14, 0.31); daprodustat vs. vadadustat, 0.09 g/dL (−0.04, 0.21); roxadustat vs. vadadustat, 0.00 g/dL (−0.22, 0.22)] or risk of MACE [all patients analysis (total *n* = 7959): daprodustat vs. roxadustat, hazard ratio (HR) 1.16 (95% CrI 0.76, 1.77); daprodustat vs. vadadustat, 0.88 (0.71, 1.09); roxadustat vs. vadadustat, 0.76 (0.50, 1.16)]. Daprodustat showed a greater increase in SF-36 Vitality compared with roxadustat [total *n* = 4880; treatment difference 4.70 points (95% CrI 0.08, 9.31)]. In dialysis patients, Hgb change from baseline was higher with daprodustat and roxadustat compared with vadadustat [all patients analysis (total *n* = 11 124): daprodustat, 0.34 g/dL (0.22, 0.45); roxadustat, 0.38 g/dL (0.27, 0.49)], while there were no clinically meaningful differences in the risk of MACE between the HIF-PHIs [all patients analysis (total *n* = 12 320): daprodustat vs. roxadustat, HR 0.89 (0.73, 1.08); daprodustat vs. vadadustat, HR 0.99 (0.82, 1.21); roxadustat vs. vadadustat, HR 1.12 (0.92, 1.37)]. Results were similar in analyses of ESA non-users and prevalent dialysis patients.

**Conclusions:**

In the setting of anaemia in CKD, indirect treatment comparisons suggest that daprodustat, roxadustat, and vadadustat are broadly clinically comparable in terms of efficacy and cardiovascular safety (precision was low for the latter), while daprodustat may be associated with reduction in fatigue to a greater extent than roxadustat.

## Graphical Abstract

**Graphical Abstract:**
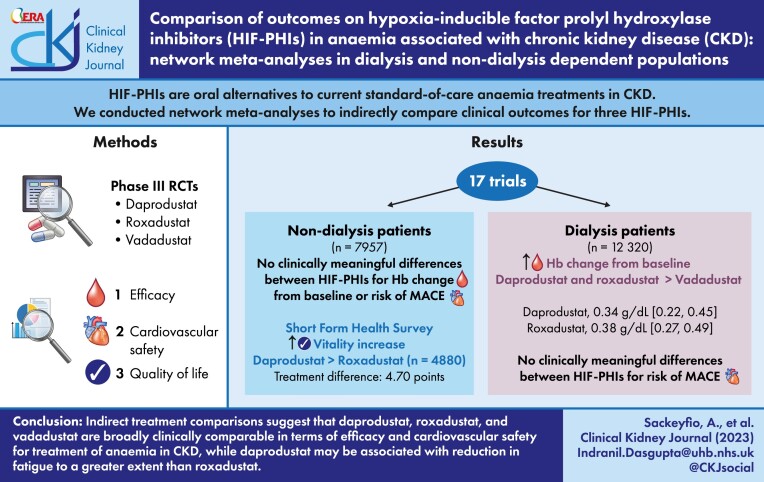

KEY LEARNING POINTS **What was known**:

- Daprodustat, roxadustat, and vadadustat are new oral medicines being developed to treat anaemia in patients with chronic kidney disease. No clinical trials have directly compared these drugs.

**This study adds**:

- We used data from separate trials to indirectly compare these drugs for their effects on haemoglobin level (effectiveness), risk of cardiovascular side-effects (safety), and fatigue (quality of life). We did this in patients on kidney dialysis and patients not needing dialysis. In non-dialysis patients, we found no differences between drugs for changes in haemoglobin or risk of cardiovascular side-effects, but daprodustat reduced fatigue more than roxadustat. In dialysis patients, changes in haemoglobin were higher with daprodustat and roxadustat over vadadustat, but there were no differences between drugs in cardiovascular side-effects.

**Potential impact**:

- These results suggest that the three drugs have similar effectiveness and safety in patients with chronic kidney disease and anaemia, but that daprodustat may be better than roxadustat for reducing fatigue.

## INTRODUCTION

Anaemia is a common complication among patients with chronic kidney disease (CKD) [1, 2]; the prevalence and severity increases with progressive kidney function decline and is associated with adverse outcomes [3–5]. Patients with anaemia in CKD often experience fatigue, shortness of breath and low exercise tolerance, and functional and cognitive impairment, affecting quality of life [2, 4, 6].

The current standard-of-care treatment for anaemia in CKD is a combination of iron supplementation and erythropoiesis-stimulating agents (ESAs), administered as infusions or injections, with the goal of increasing haemoglobin (Hgb) levels and reducing the risk of red blood cell transfusions. Hypoxia-inducible factor prolyl hydroxylase inhibitors (HIF-PHIs) are a new class of oral alternatives to conventional ESAs for treating anaemia in CKD [7, 8]. HIF-PHIs stimulate endogenous erythropoietin synthesis and may additionally affect the regulation of iron uptake, mobilisation, and transport, the production of red cells, and could even prolong red blood cell lifespan [7, 9–11]. In addition to their different mechanism of action from injectable ESAs, HIF-PHIs offer a beneficial alternative based on their oral administration, lack of need for cold-chain storage and transport, and potential to reduce the need for intravenous iron therapy [12–14]. Importantly, HIF-PHIs provide another treatment option for patients to treat their anaemia in CKD.

At least six HIF-PHIs are currently in development [8], of which three have global clinical development programmes: daprodustat, roxadustat, and vadadustat [15]. Stabilisation of HIF and stimulation of endogenous erythropoietin synthesis is one of many recognised class effects of HIF-PHIs, but they differ in molecular structure, giving rise to different pharmacokinetic and pharmacodynamic properties, dosing regimens, and potential drug–drug interactions. The HIF-PHIs inhibit different prolyl hydroxylase domain (PHD) isoforms: daprodustat preferentially inhibits PHD3 and PHD1, vadadustat inhibits PHD3, and roxadustat is a pan-PHD inhibitor [7, 16, 17]. The half-life of each agent also differs, being approximately: 3 h for daprodustat [18], 10 h for roxadustat [19, 20], and 5 h for vadadustat [16, 21], among healthy individuals. Dosing regimens for the HIF-PHIs reflect the differing characteristics of these molecules: daprodustat can be administered once-daily or three-times weekly, roxadustat three-times weekly only, and vadadustat once-daily only. They may also differ in potential drug–drug interactions (e.g. roxadustat interacts with commonly used phosphate binders [22–25]), in erythropoietic potency for the selected dose (proposed to explain improvement in Hgb and iron biomarker levels, independent of inflammation, with roxadustat vs. ESAs [26]), and in their influence on optimising iron metabolism (improved iron utilisation for daprodustat vs. ESAs [27]) or iron uptake (increased mean corpuscular-volume and -Hgb have been reported for vadadustat vs. ESAs [28]). Inherent differences in the characteristics of HIF-PHIs may therefore impact on clinical effects.

Phase III clinical trials have been completed for all three aforementioned agents in dialysis and non-dialysis populations with CKD, demonstrating non-inferiority compared with ESAs for correction of anaemia [29–38]. However, there have been no head-to-head randomised trials comparing these HIF-PHIs. In the absence of such trials, indirect treatment comparisons may provide valuable comparative evidence based on publicly available information for outcomes such as efficacy (including quality of life) and safety, to inform treatment practices [39, 40].

In this study, we conducted network meta-analyses to indirectly compare efficacy, cardiovascular safety, and quality of life (where available) between the HIF-PHIs daprodustat, roxadustat, and vadadustat used in the treatment of anaemia in CKD. Analyses were conducted separately in the non-dialysis and dialysis-dependent patient populations.

## MATERIALS AND METHODS

This article is in accordance with the Preferred Reporting Items for Systematic Reviews and Meta-Analysis (PRISMA) extension statement for reporting of systematic reviews incorporating network meta-analyses [41]. The review was not registered in any of the systematic review registries.

### Evidence base

The evidence base for this network meta-analysis study comprised phase III, randomised, controlled trials in the non-dialysis and dialysis populations of patients with anaemia in CKD that evaluated daprodustat, roxadustat, or vadadustat. Trials were identified from a systematic literature review that was performed to identify relevant citations from the published literature using searches of EMBASE, OVID MEDLINE, MEDLINE IN-PROCESS DATABASE (via PubMed), and Cochrane Central. Searches were run from database inception to 10 April 2022. These were complemented by grey literature searches, and back-referencing of relevant review articles and published congress abstracts (2016–2022). The final list of relevant trials/publications was selected from identified records after an initial assessment of relevance by screening of publication title and abstract, followed by review of full-text articles. Data were extracted from relevant publications by two independent reviewers, with discrepancies resolved by a third reviewer. At the title/abstract screening stage, both reviewers agreed on 97.6% of the publications to be included and the third reviewer resolved the discrepancy for the remaining 2.4%. At the full-text search stage, the third reviewer resolved discrepancy for 1.3% of publications to be included. For this network meta-analysis study, the key outcome data extracted were: (i) change from baseline in Hgb level for efficacy; (ii) time to first major adverse cardiovascular event (MACE) for cardiovascular safety; and (iii) change from baseline in 36-Item Short Form Health Survey (SF-36) Vitality score for quality of life. Further details of the literature searches are provided in the Supplementary Material (Supplementary Methods; PRISMA and study selection diagrams are illustrated in Supplementary Figs S1 and S2; key inclusion criteria are provided in Table S1, see online supplementary material).

Conventional indirect treatment comparisons, such as network meta-analyses, attempt to mimic the results that would be generated if all treatments had been evaluated in a single randomised trial. Network meta-analyses are an extension of traditional meta-analyses (where all included studies compare the same intervention with the same comparator) that include multiple pairwise comparisons across a range of different interventions. If trials have at least one intervention in common with one another, it is possible to develop a connected network of trials, which allows for indirect comparisons of interventions not studied in a head-to-head fashion using a network meta-analysis approach. However, for a network meta-analysis to produce unbiased results, there should not be meaningful differences across trials in effect-modifying variables (i.e. variables that alter the relative effect of a treatment, such that the treatment is more or less effective than an alternative treatment, depending on the level of the variable).

A feasibility assessment for conducting these network meta-analyses was performed by reviewing the evidence base (separately for the non-dialysis and dialysis populations) in terms of trial design, patient population, treatment implementation, and outcome definitions. Special focus was paid to characteristics that had the potential to be effect-modifying variables for any of the outcomes [42]. Further details are provided in the Supplementary Methods (see online supplementary material).

### Outcomes

For the non-dialysis population, the efficacy outcome of interest was the change from baseline in Hgb levels (g/dL). For the daprodustat trials, Hgb change from baseline was relative to the average across Week 28–52, for the roxadustat trials Hgb change from baseline was to the average across Week 28–36, and for the vadadustat trials Hgb change from baseline was to the average across Week 24–36. The cardiovascular safety outcome evaluated in the network meta-analyses was the time to first MACE, reported as a hazard ratio (HR), and defined as a composite of all-cause mortality, non-fatal myocardial infarction, and non-fatal stroke in all trials evaluating the three HIF-PHIs. Quality of life was assessed using aggregate data for pre-specified SF-36 Vitality score endpoints: change from baseline evaluated to Week 28 for the daprodustat trials and change from baseline to the average across Weeks 12–28 for the roxadustat trials (vadadustat was not included in the SF-36 Vitality network meta-analysis as no data were identified). The SF-36 Vitality score has high clinical relevance to the population with anaemia in CKD. It captures differences in subjective well-being related to energy and fatigue and has demonstrated internal consistency, reliability, and validity in patients with anaemia in CKD [43, 44].

Outcomes evaluated in the dialysis population were equivalent to those for the non-dialysis population: efficacy was assessed as Hgb change from baseline (g/dL) to the average across Week 28–52 in the daprodustat and roxadustat trials and to the average across Week 40–52 in the vadadustat trials, and cardiovascular safety was assessed as the time to first MACE, defined as stated above, for all trials included in the dialysis population network meta-analysis.

The specific populations in which analyses were carried out are indicated in Tables S2–S4 (see online supplementary material) along with their definitions. Several other efficacy and safety outcomes were considered for the network meta-analyses. These included the percentage of Hgb responders, percentage of time within the target Hgb range, use of iron supplementation, and an expanded MACE definition. With the available evidence base, robust analyses of these other outcomes were not considered feasible, as we discuss in the Supplementary Methods (see online supplementary material).

### Statistical analyses

We conducted network meta-analyses that included all patients/trials deemed likely to satisfy the assumptions required to produce unbiased estimates with a network meta-analysis during the feasibility assessment. We also conducted network meta-analyses to explore if estimates were consistent when the evidence base was restricted to a more homogenous set of trials (i.e. only trials reporting for ESA non-users at baseline in the non-dialysis population and trials that included predominantly prevalent dialysis patients in the dialysis population). The network meta-analyses were conducted using Bayesian mixed treatment comparison techniques as described in the National Institute for Health and Care Excellence Decision Support Unit Technical Support Document 2 [45]. Bayesian Markov Chain Monte Carlo (MCMC) methods with non-informative priors were used to estimate the posterior probability distribution and generate pairwise comparisons of treatments by outcome. A fixed-effects model for continuous outcomes (mean change from baseline for Hgb and SF-36 Vitality, and log HRs for MACE) with non-informative priors was utilised. Random effects models were not considered appropriate because the results would be sensitive to the specification of the heterogeneity prior (given the limited number of trials included). All analyses were implemented using the statistical software R and JAGS (‘Just Another Gibbs Sampler’, a software package for implementing MCMC techniques), with 10 000 burn-in iterations, a thinning factor of one, and three chains each with 50 000 posterior iterations. All parameters reported are the medians of the posterior distributions, together with 2.5% and 97.5% centiles to form 95% credible intervals (CrI).

## RESULTS

### Non-dialysis population evidence base

**Figure 1:**
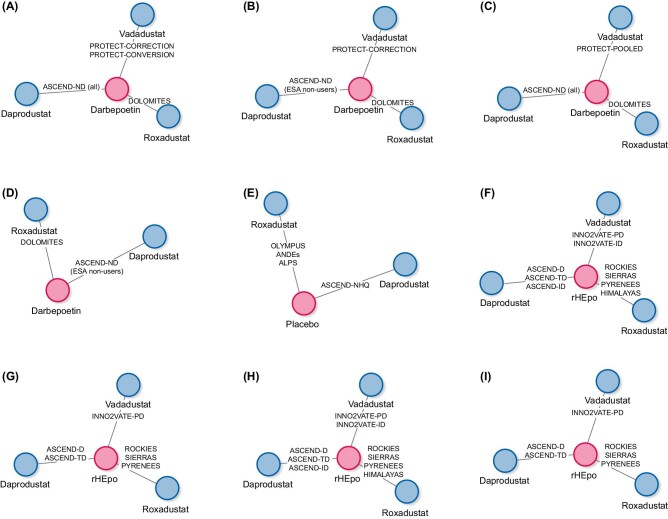
Evidence networks used in the analysis of efficacy, cardiovascular safety, and quality of life in the non-dialysis and dialysis populations. Evidence network for efficacy in the non-dialysis ESA user and ESA non-user population (**A**), efficacy in the non-dialysis ESA non-user only population (**B**), cardiovascular safety in the non-dialysis ESA user and ESA non-user population (**C**), cardiovascular safety in the non-dialysis ESA non-user only population (**D**), quality of life in the non-dialysis population (**E**), efficacy in the prevalent and incident dialysis population (**F**), efficacy in the prevalent dialysis population (**G**), cardiovascular safety in the prevalent and incident dialysis population (**H**), and cardiovascular safety in the prevalent dialysis population (**I**). ESA, erythropoiesis-stimulating agent; rHEpo, recombinant human erythropoietin.

The evidence base for the non-dialysis population of patients with anaemia in CKD comprised eight trials, four of which contributed to the network meta-analyses of Hgb change from baseline and MACE, and the other four to the network meta-analysis of change from baseline in SF-36 Vitality. Details of the included trials are provided in Tables S2 and S3, and baseline characteristics detailed in Table S5 (see online supplementary material). Placebo-controlled trials were excluded for efficacy and CV outcome analyses as differences in treatment target range and/or dosing across trials are expected to modify treatment effects for efficacy- and safety-related outcomes when all treatment arms were compared with placebo; whereas, in active-controlled trials, these differences were not expected to lead to modifications of treatment effects as both treatment arms are impacted in a similar way within each trial (reasons why other trials were excluded from analyses are documented in the Supplementary Methods, see online supplementary material). The analyses of Hgb and MACE both included one trial with daprodustat (ASCEND-ND), one trial with roxadustat (DOLOMITES) and two trials with vadadustat (PRO2TECT CORRECTION and PRO2TECT CONVERSION), all with darbepoetin as the comparator. The daprodustat trial and both vadadustat trials were event-driven cardiovascular outcome trials; the roxadustat trial was not. The roxadustat trial included only ESA non-users at baseline, the daprodustat trial included both ESA users and non-users at baseline, whereas for vadadustat separate trials were conducted in ESA non-users (PRO2TECT CORRECTION) and ESA users (PRO2TECT CONVERSION). Hgb and MACE results within each ESA group (users and non-users) were available from the daprodustat trial. Only pooled results from the two vadadustat trials (combining the ESA user and non-user populations) were reported for MACE. The analysis of SF-36 Vitality included one trial with daprodustat (ASCEND-NHQ) and three trials with roxadustat (ALPS, ANDES, and OLYMPUS), each compared with placebo. The daprodustat and vadadustat trials evaluated once-daily treatment, and the roxadustat trials three-times weekly treatment. Aggregate trial-level results for each HIF-PHI relative to comparator that were used as inputs for the network meta-analyses in the non-dialysis population are presented in Table S6 (see online supplementary material). The evidence network for each meta-analysis is depicted in Fig. 1A–E.

### Dialysis population evidence base

For the dialysis population of patients with anaemia in CKD, the evidence base comprised a total of nine trials, all contributing to the network meta-analyses of Hgb change from baseline and MACE. Details of the trials are provided in Table S4, and baseline characteristics detailed in Table S7 (reasons why other trials were excluded from analyses are documented in the Supplementary Methods), see online supplementary material. The included trials were all active controlled: ASCEND-D, ASCEND-TD, and ASCEND-ID for daprodustat; ROCKIES, SIERRAS, PYRENEES, and HIMALAYAS for roxadustat; and INNO2VATE-PD and INNO2VATE-ID for vadadustat. One daprodustat trial (ASCEND-D) and both vadadustat trials were event-driven cardiovascular outcome trials but none of the roxadustat trials were designed as such. (ROCKIES was designed to ensure a prespecified target number of MACE to contribute to a pre-planned pooled cardiovascular safety analysis). The trials included prevalent dialysis patients, with the exception of ASCEND-ID, HIMALAYAS, and INNO2VATE-ID (which included solely incident dialysis patients) and ROCKIES and SIERRAS (20% and 10% incident dialysis patients, respectively). The ASCEND-TD trial evaluated daprodustat as a three-times weekly treatment, as did all four trials with roxadustat, whereas once-daily treatment was used in the ASCEND-D and ASCEND-ID daprodustat trials and both vadadustat trials. Aggregate trial-level results for each HIF-PHI relative to comparator that were used as inputs for the network meta-analyses in the dialysis population are presented in Table S8 (see online supplementary material). The evidence network for each meta-analysis is depicted in Fig. 1F–I.

### Non-dialysis population network meta-analyses

#### Efficacy

**Figure 2:**
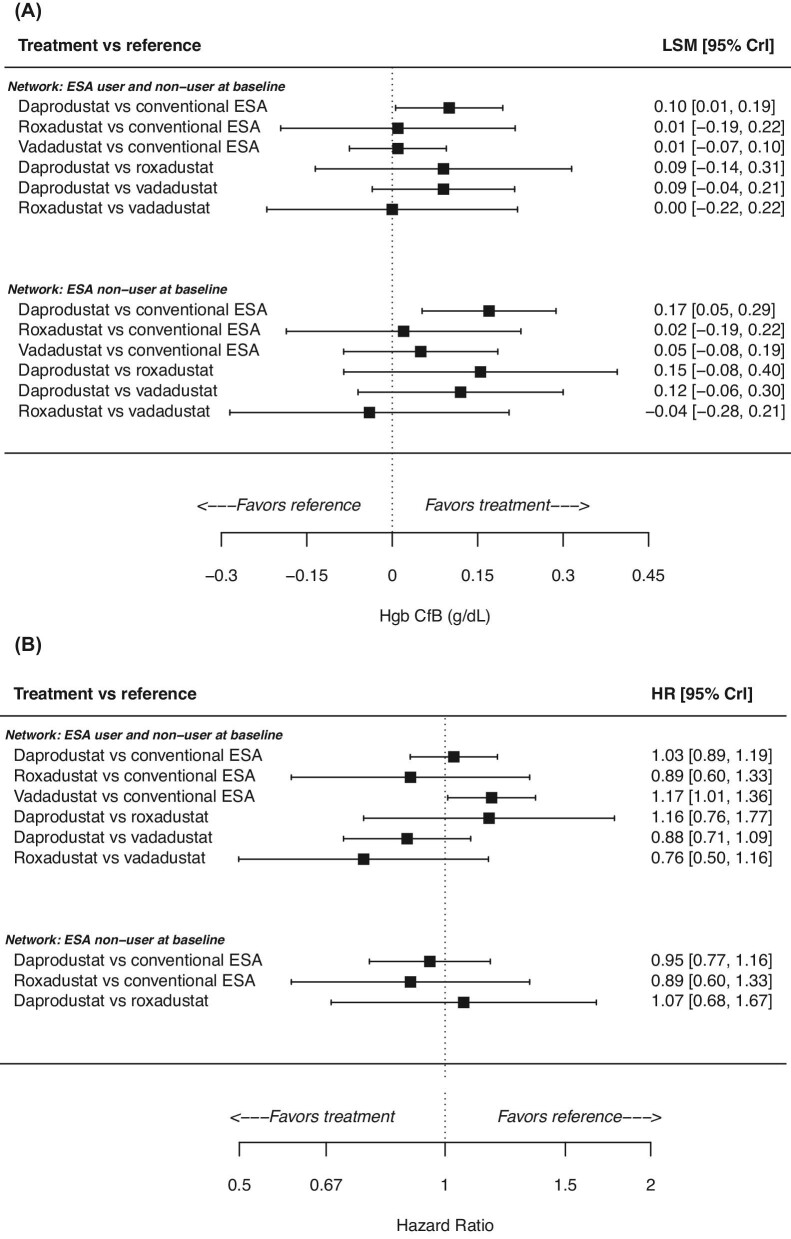
Efficacy (**A**) and cardiovascular safety (**B**) of daprodustat, roxadustat, and vadadustat in the non-dialysis population. CfB, change from baseline; CrI, credible interval; ESA, erythropoiesis-stimulating agent; Hgb, haemoglobin.

For the analysis that included patients regardless of ESA user status at baseline, relative to darbepoetin, patients who received daprodustat, roxadustat, or vadadustat showed a small change from baseline in the median of the posterior distribution for Hgb estimated from the fixed-effects network meta-analysis (Fig. 2A). Observed differences between the HIF-PHIs were also small: the difference (95% CrI) in Hgb change from baseline for daprodustat vs. roxadustat was 0.09 g/dL (−0.14, 0.31), for daprodustat vs. vadadustat was 0.09 g/dL (−0.04, 0.21), and for roxadustat vs. vadadustat was −0.00 g/dL (−0.22, 0.22) (Fig. 2A).

Since all patients in the roxadustat trial were ESA non-users, a separate network meta-analysis was conducted that included only patients who were ESA non-users at baseline. Results were similar to the initial analysis: Hgb change from baseline relative to darbepoetin was small for all three HIF-PHIs (Fig. 2A), as were the differences (95% CrI) in Hgb change from baseline between the three HIF-PHIs: daprodustat vs. roxadustat: 0.15 g/dL (−0.08, 0.40); daprodustat vs. vadadustat: 0.12 g/dL (−0.06, 0.30); roxadustat vs. vadadustat: −0.04 g/dL (−0.28, 0.21) (Fig. 2A).

#### MACE

For the analysis that included patients regardless of ESA user status at baseline, the median follow-up was approximately 1.7–2.1 years across the trials. The HR (95% CrI) for MACE was higher for patients who received vadadustat relative to darbepoetin (HR 1.17; 1.01, 1.36) and was similar to darbepoetin for patients who received daprodustat and roxadustat (Fig. 2B). The risk of MACE between the three HIF-PHIs [HR (95% CrI)] was: daprodustat vs. roxadustat 1.16 (0.76, 1.77); for daprodustat vs. vadadustat 0.88 (0.71, 1.09); and for roxadustat vs. vadadustat 0.76 (0.50, 1.16) (Fig. 2B).

In the analysis that included only patients who were ESA non-users at baseline, the HR (95% CrI) of MACE was similar to darbepoetin for daprodustat (HR 0.95; 0.77, 1.16) and roxadustat (HR 0.89; 0.60, 1.33) (Fig. 2B), and similar when comparing daprodustat and roxadustat (HR 1.07; 0.68, 1.67) (Fig. 2B). Vadadustat was not included because only pooled MACE data were available across the ESA non-user and ESA user sub-groups.

#### Quality of life: SF-36 Vitality

**Figure 3:**
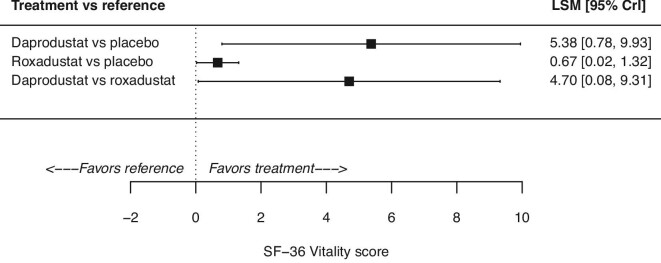
Quality of life assessed as the SF-36 Vitality score for daprodustat and roxadustat in the non-dialysis population. CrI, credible interval.

Relative to placebo, the difference (95% CrI) in SF-36 Vitality score was 5.38 points (0.78, 9.93) for daprodustat and 0.67 points (0.02, 1.32) for roxadustat (Fig. 3). The observed increase was greater among patients treated with daprodustat when compared with patients who received roxadustat (4.70 points; 0.08, 9.31) (Fig. 3).

### Dialysis population network meta-analyses

#### Efficacy

**Figure 4:**
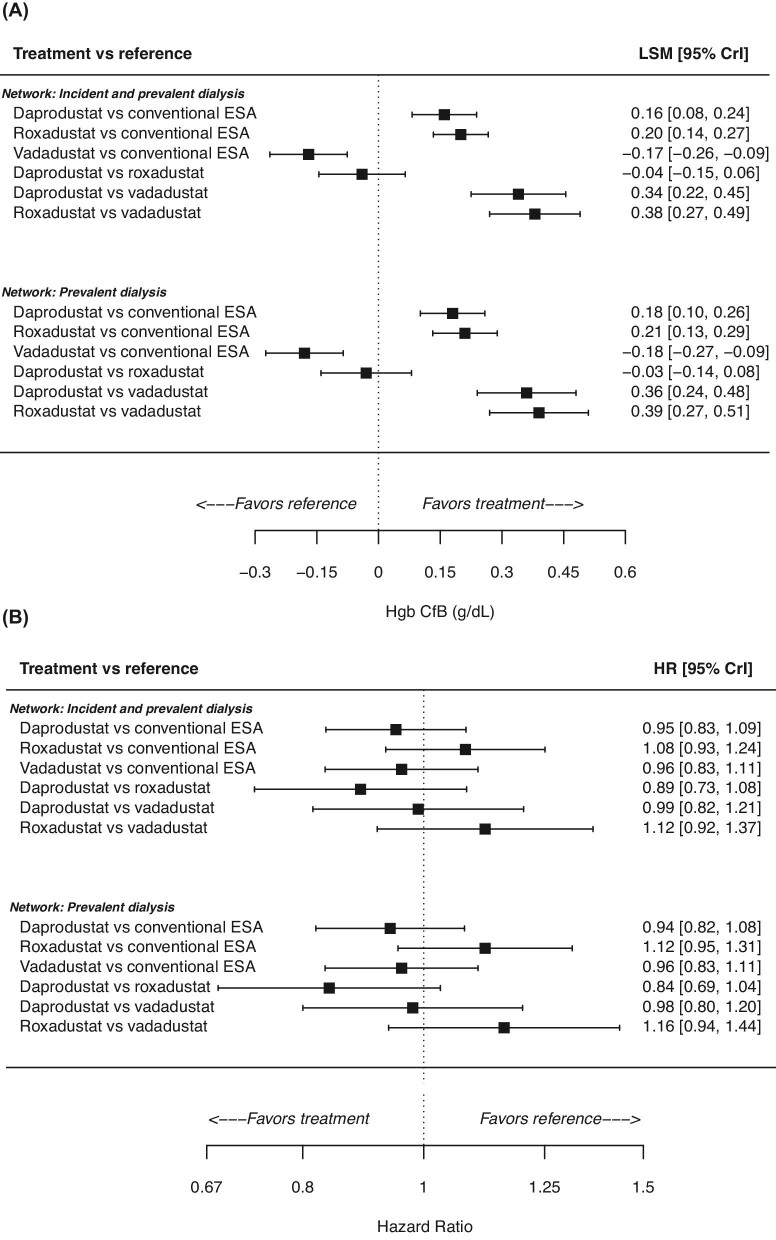
Efficacy (**A**) and cardiovascular safety (**B**) of daprodustat, roxadustat, and vadadustat in the dialysis population. CfB, change from baseline; CrI, credible interval; ESA, erythropoiesis-stimulating agent; Hgb, haemoglobin.

For the analysis that included both incident and prevalent dialysis trials, the median (95% CrI) of the posterior distribution for Hgb change from baseline was greater relative to darbepoetin/epoetin for patients who received daprodustat (0.16 g/dL; 0.08, 0.24) and roxadustat (0.20 g/dL; 0.14, 0.27), but smaller for patients who received vadadustat (−0.17 g/dL; −0.26, −0.09) (Fig. 4A). The difference (95% CrI) in Hgb change from baseline for daprodustat vs. roxadustat was small (−0.04 g/dL; −0.15, 0.06), but both showed a larger change compared with vadadustat (daprodustat: 0.34 g/dL; 0.22, 0.45; roxadustat: 0.38 g/dL; 0.27, 0.49) (Fig. 4A).

Results were similar in the analysis that excluded trials solely in incident dialysis patients. Relative to darbepoetin/epoetin, daprodustat and roxadustat showed a greater Hgb change from baseline whereas vadadustat showed a decrease (Fig. 4A). Comparing the three HIF-PHIs, larger changes in Hgb from baseline were observed for daprodustat (0.36 g/dL; 0.24, 0.48) and roxadustat (0.39 g/dL; 0.27, 0.51) compared with vadadustat (Hgb change from baseline was similar between daprodustat and roxadustat: −0.03 g/dL; −0.14, 0.08) (Fig. 4A).

#### MACE

For the analysis that included both incident and prevalent dialysis trials, duration of follow-up was as follows: for daprodustat, median follow-up was 2.5 years in ASCEND-D (not reported for ASCEND-ID and ASCEND-TD; both studies were of 1-year duration, plus 4–6 weeks follow-up); for roxadustat, the study duration ranged from 1–4 years (plus 4 weeks follow-up); and for vadadustat, median follow-up was 1.7 years in INNO2VATE-PD and 1.2 years in INNO2VATE-ID. There was no clear difference in HR of MACE relative to darbepoetin/epoetin for each HIF-PHI (Fig. 4B). Similarly, differences were small in comparison between the three HIF-PHIs: HR (95% CrI) for daprodustat vs. roxadustat was 0.89 (0.73, 1.08); for daprodustat vs. vadadustat it was 0.99 (0.82, 1.21); and for roxadustat vs. vadadustat it was 1.12 (0.92, 1.37) (Fig. 4B).

In an analysis that excluded the trials conducted solely in incident dialysis patients, there were no clear differences in the risk of MACE for the HIF-PHIs relative to darbepoetin/epoetin (Fig. 4B) or to each other (daprodustat vs. roxadustat: HR 0.84; 0.69, 1.04; daprodustat vs. vadadustat: HR 0.98; 0.80, 1.20; roxadustat vs. vadadustat: HR 1.16; 0.94, 1.44) (Fig. 4B).

## DISCUSSION

These network meta-analyses of phase III, randomised, controlled trials provide comparative outcome data for the three HIF-PHIs with global development programs. In non-dialysis-dependent CKD patients, daprodustat, roxadustat, and vadadustat were comparable with respect to efficacy (change in Hgb from baseline). In terms of cardiovascular safety (time to first MACE) in this population, risk of MACE was higher with vadadustat relative to ESA but there were no clear differences in risk when comparing the three HIF-PHIs. Also in non-dialysis CKD patients, daprodustat and roxadustat showed a greater effect on quality of life (SF-36 Vitality change from baseline) relative to placebo, with the difference greater for daprodustat than for roxadustat. In dialysis-dependent CKD patients, daprodustat and roxadustat demonstrated greater changes in efficacy compared with both ESA and vadadustat, but there were no clear differences between the three HIF-PHIs in cardiovascular safety.

The results were consistent across network analyses that included all patients/trials or when the evidence base was restricted to a more homogenous set of trials. This suggests that the findings are robust in different patient sub-groups (ESA users and ESA non-users) and dialysis types (prevalent dialysis and incident dialysis). The 95% CrI around point estimates of time to MACE were generally wide, especially in the non-dialysis population, indicating low precision and a degree of uncertainly that prohibits definitive conclusions regarding differences in cardiovascular safety among the three HIF-PHIs. This was not the case for Hgb efficacy, however, where comparisons among the three HIF-PHIs had relatively high levels of precision.

Our analyses in non-dialysis-dependent patients revealed that the three HIF-PHIs had similar and minimal effects on change in Hgb relative to ESA (highest for daprodustat). Similarly, differences in effects on Hgb among daprodustat, roxadustat, and vadadustat were small and comparable. These findings were consistent in the analysis of all patients/trials and when the analysis included only ESA non-users. Evidence from our indirect comparisons may not support a clinically meaningful difference in efficacy among the agents. Concerning cardiovascular safety, the individual phase III clinical trials with daprodustat [37] and roxadustat [29] showed these to be non-inferior to darbepoetin for the risk of MACE in non-dialysis patients, whereas vadadustat [31] failed to meet non-inferiority for this cardiovascular endpoint. The results of our network analyses are consistent with this. The indirect treatment comparisons among the three HIF-PHIs identified no clear differences between the agents for cardiovascular safety. However, it is difficult to gauge the clinical relevance of the results; we may have to wait for results of randomised controlled trials comparing the HIF-PHI agents head-to-head, or until substantial real-world data are available in the future. Although the results were similar in the separate analyses of all patients and ESA non-users, the analyses of MACE included a single trial with roxadustat (DOLOMITES) that was done in a European population with a small sample size and was not an event-driven cardiovascular outcome trial (unlike the single daprodustat and two vadadustat global trials included). The roxadustat trial had a length of follow-up comparable with the daprodustat trial, and the lower number of events in the former resulted in a point estimate with low precision (wide CrI), which limits interpretation of the long-term safety endpoint.

We evaluated comparative effects of daprodustat and roxadustat on quality of life in the non-dialysis population using the SF-36 Vitality score. In a network meta-analysis including randomised placebo-controlled trials, both daprodustat and roxadustat treatment resulted in larger changes from baseline in SF-36 Vitality than placebo; however, the magnitude of effect observed in the individual trials was considerably different: treatment difference of 5.36 for daprodustat [46] compared with 1.13, 1.22, and 0.44 for the three trials with roxadustat [47]. In the trial with daprodustat (ASCEND-NHQ), the change from baseline in SF-36 Vitality score was 7.3 points, which met the predetermined threshold of six points [46], indicating the change was clinically meaningful by improving vitality and decreasing fatigue in patients with anaemia in CKD when treated to a Hgb target of 11–12 g/dL (for comparison, the roxadustat trials had Hgb targets of ≥11 g/dL, 11 ±1 g/dL, and 10–12 g/dL). The timeframe for the SF-36 Vitality endpoint in the trials, and thus our analyses, differed since data at Week 28 were unavailable for roxadustat (analysed as the average across Weeks 12–28, versus to Week 28 for daprodustat). However, this difference was relatively small and would not be expected to significantly impact the treatment effect. Our analyses show that daprodustat resulted in a considerably greater change in SF-36 Vitality than did roxadustat, with a potentially clinically meaningful difference of 4.70 (based on a minimal clinically important difference of 3–5 points) [48, 49]. A similar network meta-analysis in the dialysis population was not feasible because patients who are dialysis-dependent typically require treatment with an ESA, such that a placebo arm is not typical for trials in this population.

Unlike in the non-dialysis patients, there were differences between the agents in the dialysis population. Both daprodustat and roxadustat treatment led to small changes in Hgb levels relative to ESA and changes of approximately 0.3–0.4 g/dL more than with vadadustat. This was evident in the analysis that included both prevalent and incident dialysis patients and also when excluding the incident dialysis population. In the current analyses, there was a difference in the time-period over which Hgb was evaluated in the trials with the different HIF-PHIs: Week 28–52 for daprodustat and roxadustat, and Week 40–52 for vadadustat. However, this difference was relatively small and would not be expected to impact the treatment effect. This said, the potential difference in efficacy we observed for daprodustat and roxadustat compared with vadadustat in dialysis-dependent patients would ideally be confirmed in suitably designed head-to-head trials that also could establish the clinical relevance of any potential difference (the individual trials of daprodustat and roxadustat versus ESA used a non-inferiority margin of −0.75 g/dL), as the difference in efficacy among HIF-PHI molecules is also influenced by the relative potency of the dose chosen. Alternatively, analysis of large, meticulously collected, real-world data may also help to address this. The risk of MACE in dialysis-dependent patients was comparable among the three HIF-PHIs. Evidence from cardiovascular outcome trials has previously demonstrated non-inferiority of daprodustat and vadadustat relative to darbepoetin with respect to cardiovascular safety in the dialysis setting [34, 38].

Although meta-analyses of multiple HIF-PHIs for treating anaemia in CKD have been reported [50–55], some of which included cardiovascular outcome trials in the analyses, to our knowledge, these are the first network meta-analyses in both non-dialysis and dialysis populations comparing three globally developed HIF-PHIs. The indirect treatment comparisons we conducted in patients with anaemia in CKD focused on important clinical outcomes of efficacy (Hgb change from baseline), cardiovascular safety (risk of MACE), and quality of life (SF-36 Vitality score). However, our work has limitations, such as the wide credible interval in our quality-of-life findings. Common to all meta-analyses, it is possible that cross-trial differences in trial design and patient characteristics may have modified the treatment effect and introduced bias in the comparisons we report. The analyses we performed assumed that characteristics such as age, race, sex, body mass index, stage of CKD, baseline Hgb, entry iron status, permitted concomitant treatments, and geographic region were not relative treatment effect modifiers. If one or more of these assumptions is not valid, this could introduce a degree of bias to the comparisons. Other limitations include that the roxadustat trials also enrolled iron deficient patients and the difference in treatment target range for Hgb across the daprodustat, roxadustat, and vadadustat trials, which may impact the dosing scheme and observed effects of treatment on the outcomes evaluated and effect on quality of life.

Furthermore, several potential outcomes were infeasible, including the percentage of responders (mean Hgb in the target range), the percentage of time within the target Hgb range, the need for intravenous iron supplementation, MACE+ as a further cardiovascular safety outcome, and mortality (all-cause and cardiovascular). This is because the evidence base identified did not allow robust analyses of these (see Supplementary Material, see online supplementary material). For the percentage of responders, this was because of different definitions for a Hgb response between trials with the different HIF-PHIs and also between trials with the same HIF-PHI, with differences also between regions in some trials. Similarly, evaluating MACE+ as a safety outcome was not feasible because of differences in the definition used across the trials: the daprodustat and vadadustat trials defined MACE+ as MACE plus hospitalisation for either heart failure or a thromboembolic event (and excluding vascular access failure for the vadadustat trials), whereas a definition of MACE plus unstable angina or congestive heart failure requiring hospitalisation was used in the roxadustat trials. The heterogenous reporting across trials of individual components of MACE and non-cardiovascular adverse events, which would require multiple adjustments of data prior to any analyses, did not support indirect treatment comparisons of these outcomes.

In conclusion, these network meta-analyses provide comparative outcome data for the three HIF-PHIs with global development programs in non-dialysis and dialysis-dependent CKD patients. In non-dialysis CKD patients, daprodustat, roxadustat, and vadadustat were clinically comparable with respect to efficacy and cardiovascular safety (although precision was low for the analyses of MACE), whereas daprodustat seems to be associated with a greater benefit on quality of life compared with roxadustat. In dialysis-dependent CKD patients, daprodustat and roxadustat were associated with a greater change over vadadustat in terms of efficacy, whereas the three HIF-PHIs were clinically comparable for cardiovascular safety (albeit with low precision). Head-to-head randomised trials in the future will be needed to confirm the results. Well designed, observational studies based on large, meticulously collected, real-world data may also help to address some of the questions raised. Until then, these results may inform the decision-making process of regulatory authorities, clinicians, health technology agencies, payers, and health service commissioners.

## Supplementary Material

sfad298_Supplemental_Files

## Data Availability

Information on GSK's data sharing commitments and requests for access to anonymised individual participant data and associated documents can be found at www.clinicalstudydatarequest.com.
