# Supplementary material for: Comparison of outcomes on hypoxia-inducible factor prolyl hydroxylase inhibitors (HIF-PHIs) in anaemia associated with chronic kidney disease: network meta-analyses in dialysis and non-dialysis dependent populations
Source: Clin Kidney J. 2023 Dec 7;17(1):sfad298. doi: 10.1093/ckj/sfad298 (PMC10799328; doi:10.1093/ckj/sfad298)
Supplement: sfad298_Supplemental_Files [file sfad298_supplemental_files.zip › Suppl_Indirect Treatment Comparisons_AMC_31Aug23.docx]

**Supplementary Material**

**Supplementary Methods**

***Systematic literature review***

The evidence base for the network meta-analyses was compiled from a systematic literature review that was performed and included the identification of published literature reporting results of phase III, randomised, controlled trials with daprodustat, roxadustat, or vadadustat in the non-dialysis and dialysis patient populations with anaemia in chronic kidney disease (CKD). Relevant citations from the published literature were identified through structured searches of the following key biomedical literature databases: (1) Medical Literature Analysis and Retrieval System Online (MEDLINE); (2) Excerpta Medica Database (Embase); (3) MEDLINE In-Process via PubMed; and (4) Cochrane Central, including the Cochrane Controlled Trials Register (CCTR) and Cochrane Database of Systematic Reviews (CDSR). Searches were run on MEDLINE and Embase using the embase.com interface, and on Cochrane Central using the Cochrane interface. Searches were run from database inception to 10 April 2022. To ensure that all relevant evidence was captured, supplementary keyword-based pragmatic searches were conducted in Google and Google Scholar, bibliographic searches of recent primary studies, systematic reviews, and general reviews were performed, as were searches of published abstracts across the period 2016–2022 from relevant congresses (World Nephrology Conference, European Nephrology Conference, Annual Kidney Congress, ISN World Congress of Nephrology [WCN], ERA-EDTA Annual Congress, American Society of Nephrology [ASN] Kidney Week, Global Nephrologists Annual Meeting, and National Kidney Foundation Conference).

A comprehensive search strategy was developed based on the population, interventions, comparators, outcomes, study type (PICOS) framework to identify relevant data from identified publications. Studies were selected in two steps: first, the title and abstract of each publication identified during the structured and supplementary searches were screened for relevance; second, full-text versions of the selected publications were reviewed to select the final list of trials to be included in the analyses. For both steps of the selection process, each publication was assessed for inclusion or exclusion by two independent reviewers, and any discrepancies were resolved by a third reviewer. Data on study characteristics, patient characteristics, and study outcomes were extracted from the selected publications/studies using a predefined Microsoft Excel-based template. Data were extracted by two independent reviewers (a third reviewer resolved any discrepancies). For this network meta-analysis study, the key outcome data were: (1) change from baseline in haemoglobin (Hgb) level for efficacy; (2) time to first major adverse cardiovascular event (MACE) for cardiovascular safety; and (3) change from baseline in Short Form Health Survey (SF-36) Vitality score for quality of life.

***Evidence base and feasibility assessment for the network meta-analyses***

The feasibility of conducting network meta-analyses was assessed by reviewing the identified evidence base (separately for the non-dialysis and dialysis populations) in terms of trial design, patient populations, treatments, and outcome definitions.

*Trial design and patients for the three outcomes evaluated with network meta-analyses*

Eligible publications identified from the systematic literature review, plus available unpublished data for daprodustat, reported data for a total of unique 17 trials that were used in one or more of the network meta-analyses. The trials were all phase III, randomised, active-controlled (13 trials) or placebo-controlled (four trials) trials in non-dialysis (eight trials) or dialysis dependent (nine trials) patients with anaemia in CKD that evaluated daprodustat (five trials), roxadustat (eight trials), or vadadustat (four trials). In the non-dialysis population, the sample size was n=614 and n=3872 for the two daprodustat trials, ranged from n=559 to n=2761 for the four roxadustat trials, and was n=1725 and n=1751 for the two vadadustat trials. In the dialysis population, the sample size ranged from n=312 to n=2964 for three daprodustat trials, from n=741 to n=2106 for the four roxadustat trials, and was n=192 and n=2526 for the two vadadustat trials. In terms of geographic location, all trials except one were international trials: of the eight trials in the non-dialysis population, both daprodustat trials, both vadadustat trials, and one roxadustat trial were worldwide, two roxadustat trials were conducted in Europe, and one roxadustat was conducted in the USA, South America, Asia, and Australia; of the nine trials in the dialysis population, all three daprodustat trials, both vadadustat trials, and two roxadustat trials were worldwide, one roxadustat trial was conducted in Europe, and one roxadustat trial in the USA.

Trials were either conducted in the non-dialysis population (eight trials) or dialysis dependent population (nine trials), with trials in the latter comprising patients with prevalent dialysis (four trials), incident dialysis (three trials) or both prevalent and incident dialysis (two trials). Three trials in the non-dialysis population and three trials in the dialysis population were event-driven cardiovascular outcome trials. Of the four active-controlled trials in the non-dialysis population, two trials included only erythropoiesis-stimulating agents (ESA) non-users, one trial included only ESA users, and one trial both ESA users and ESA non-users. Daprodustat was administered once-daily in four trials and three-times weekly in one trial. Roxadustat was administered three-times weekly in all eight trials. Vadadustat was administered once daily in all four trials.

The analyses of efficacy and cardiovascular safety outcomes in both the non-dialysis population and dialysis population excluded trials identified from the systematic literature review that were conducted exclusively in Asia. This was due to differences in standard of care between regions that could lead to effect modification concerns about generalisability of study findings. We compared trials that were conducted across many countries and populations rather than those conducted in a specific population and hence the Nangaku et al., 2021 study (daprodustat) and FGCL-4592-808 and 1517-CL-0310 trials (Chen et al., 2019 [both non-dialysis CKD and dialysis CKD]; roxadustat) were excluded during the screening phase of the systematic literature review because they were conducted among Japanese or Chinese non-dialysis patients only. Furthermore, the design of the Nangaku et al., 2021 study and FGCL-4592-808 and 1517-CL-0310 trials differed from ASCEND-ND. ASCEND-ND was a cardiovascular outcome trial, whereas the Nangaku et al., 2021 study had insufficient sample size and relatively short duration to evaluate cardiovascular events, and the FGCL-4592-808 and 1517-CL-0310 trials did not report on any cardiovascular events for comparison.

Placebo-controlled trials were also excluded from the network meta-analyses of efficacy and cardiovascular safety outcomes in the non-dialysis population. This was because of differences between the hypoxia-inducible factor prolyl hydroxylase inhibitors (HIF-PHI) trials in the treatment target range/dosing scheme that could potentially modify the efficacy and safety treatment effects when compared to placebo. The difference across trials in treatment target range/dosing scheme was not expected to lead to effect-modification in the active-controlled trials since both treatment arms are likely modified in the same way (note that only active-controlled trials were conducted in the dialysis population). For the analysis of quality of life, only placebo-controlled trials with daprodustat and roxadustat in the non-dialysis population were considered. This was due to the acceptability of placebo-controlled data to assess quality of life improvement with a new therapeutic versus no treatment.

*Clinical outcomes assessed but not considered feasible for inclusion in the analyses*

Beyond the three outcomes we did evaluate, several other outcomes were considered for inclusion in the network meta-analyses. Examples of these are listed below, together with reasons why robust analyses of these outcomes were not considered feasible based on review of the available evidence base.

Percentage of Hgb responders: The definition of a Hgb response differed between trials with the different HIF-PHIs. Examples of Hgb response definitions follow: (1) For some daprodustat trials, response was defined as a mean Hgb within the target range pf 10.0–11.5 g/dL; (2) For some roxadustat trials in non-dialysis patients, a response was defined as Hgb ≥11.0 g/dL and an increase of ≥1.0 g/dL (in patients with baseline >8.0 g/dL) or Hgb increase ≥2.0 g/dL (in patients with baseline ≤8.0 g/dL) at two consecutive visits ≥5 days apart during Weeks 1–24 without rescue therapy; the definition in dialysis patients was a mean serum Hgb of 10.0–12.0 g/dL during Weeks 28–36, without rescue therapy within 6 weeks prior to and during Weeks 28–36; and (3) For vadadustat trials, a response was defined as an average Hgb within a patient’s country-specific target range during Weeks 24–36 (and Weeks 40–52, depending on the timepoint evaluated). Further to the differences in definitions, the Hgb target differed between trials: 10–11 g/dL for daprodustat, 10–12 g/dL for roxadustat, and 10–11 g/dL (USA) or 10–12 g/dL (rest of the world) for vadadustat.

Percentage of time within the target Hgb range: This outcome was not routinely reported in the different trials of daprodustat, roxadustat, and vadadustat.

Iron supplementation: Iron supplementation/management protocols differed between the trials with the three HIF-PHIs and in some cases differed between treatment arms within a trial (e.g. the ROCKIES and HIMALAYAS trials of roxadustat).

Individual MACE components: There was heterogenous reporting between trials for individual components of the composite MACE outcome, which would require multiple data adjustments prior to any analyses.

MACE+: The definition of MACE+ differed between trials. For the daprodustat and vadadustat trials, MACE+ was defined as MACE plus hospitalisation for either heart failure or a thromboembolic event (and excluding vascular access failure for the vadadustat trials). For the roxadustat trials, MACE+ was defined as MACE plus unstable angina or congestive heart failure requiring hospitalisation.

Non-cardiovascular adverse events: There was heterogenous reporting between trials for non-cardiovascular adverse events, which would require multiple data adjustments prior to any analyses.

**Supplementary Figure 1.** Preferred Reporting Items for Systematic Reviews and Meta-Analyses (PRISMA) flow diagram.


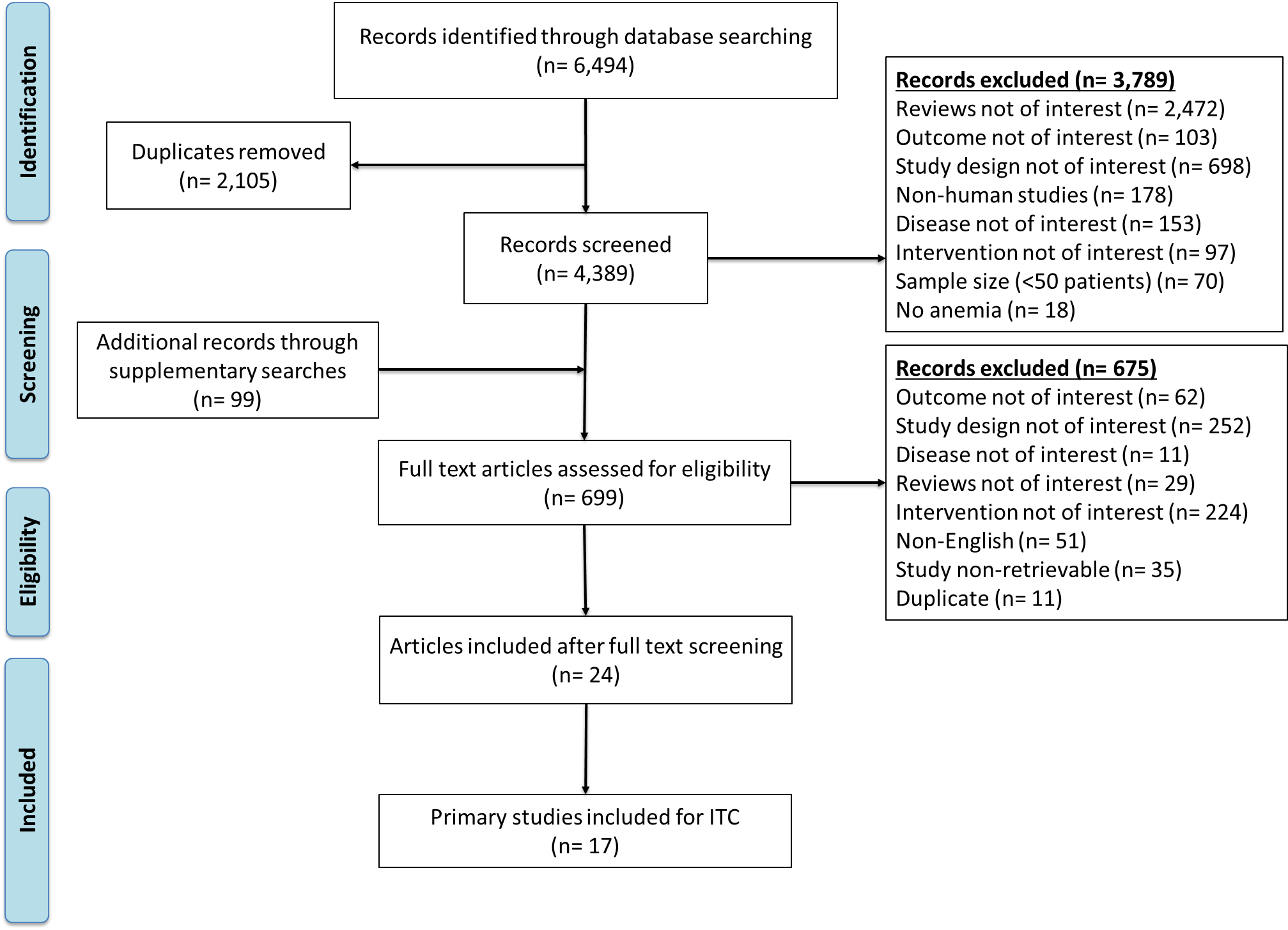

Following full-text screening of n=24 articles, n=7 articles were excluded as the studies were conducted solely in Asian populations leaving n=17 articles for inclusion in the indirect treatment comparison analysis. ITC, indirect treatment comparison.

**Supplementary Figure 2.** Flowchart for study selection


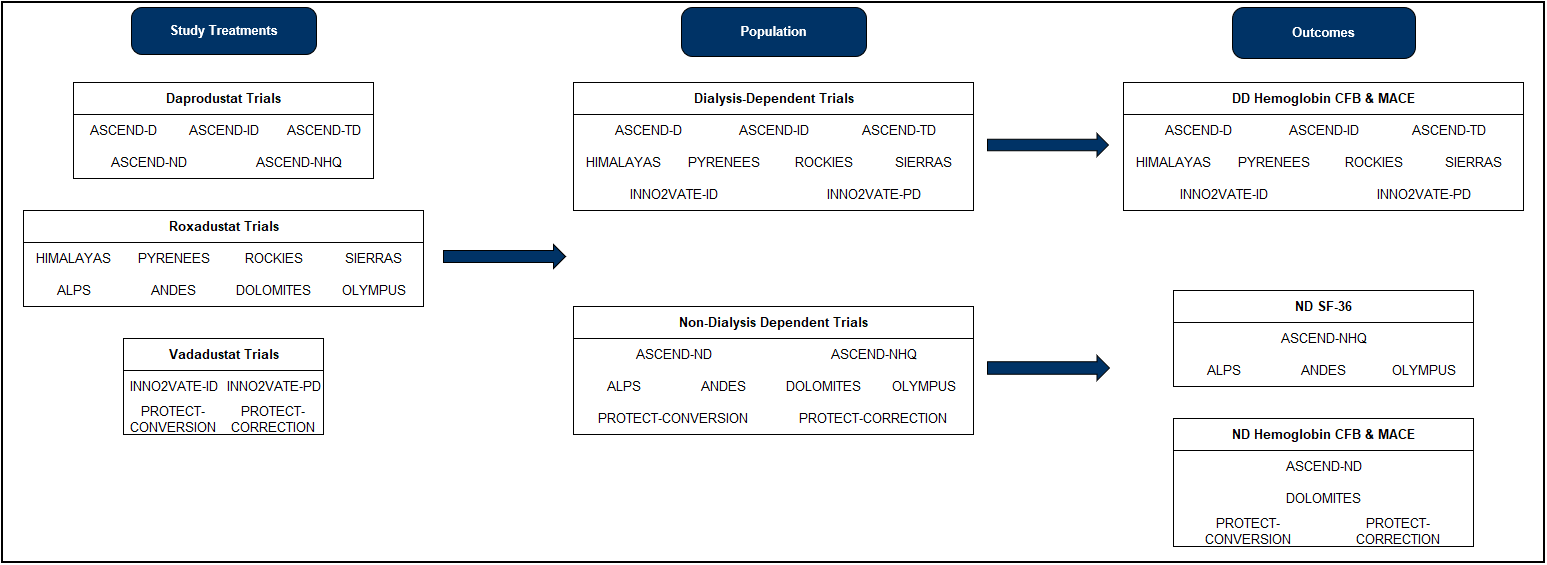


CFB, change from baseline; DD, dialysis-dependent; MACE, major adverse cardiovascular event; ND, non-dialysis; SF-36, 36-Item Short Form Health Survey.

**Supplementary Table 1.** Key inclusion criteria for trials included in the (A) non-dialysis population and (B) dialysis network meta-analyses.

(A)

| **Trials** | **Hgb entry range** | **ESA exposure** | **ESA duration** | **Reported baseline Hgb** |
| --- | --- | --- | --- | --- |
| ASCEND-ND (NCT02876835) | Group 1 (not using ESAs):  8.0–10.0 g/dL  Group 2 (ESA users):  8.0–11.0 g/dL | Both ESA  non-users  and ESA  exposed | Group 1 (not using ESAs): No ESA use within the 6 weeks prior to screening and no ESA use between screening and randomisation  Group 2: (ESA users): Use of any approved ESA for the 6 weeks prior to screening and continuing between screening and randomisation | Dapro: 9.87 g/dL  RhEpo: 9.85 g/dL |
| DOLOMITES (NCT02021318) | The mean of the 2 most recent Hgb values during the screening period, obtained at least 4 days apart: ≤10.5 g/dL, with a difference of ≤1.0 g/dL | ESA non-users | Patients who received ESA treatment within 12 weeks of randomisation were excluded | Roxa: 9.6 g/dL  RhEpo: 9.6 g/dL |
| PRO2TECT CORRECTION (NCT02648347) | <10.0 g/dL | ESA non-users | Patients who received ESA treatment within 8 weeks of randomisation were excluded | Vada: 9.1 g/dL  RhEpo: 9.1 g/dL |
| PRO2TECT CONVERSION (NCT02680574) | 1. USA: 8.0–11.0 g/dL  2. Non-USA: 9.0–12.0 g/dL | Currently maintained on  ESA therapy | Currently maintained on ESA therapy, with a dose received within 6 weeks before screening | Vada: 10.4 g/dL  RhEpo: 10.4 g/dL |
| ASCEND-NHQ  (NCT03409107) | 8.5–10.0 g/dL | ESA non-users | Patients with history of rhEPO or rhEPO analogue use within the 8 weeks prior to screening and rhEPO use between screening and randomisation were excluded | Dapro: 9.7 g/dL  Pbo: 9.7 g/dL |
| OLYMPUS  (NCT02174627) | <10.0 g/dL | ESA non-users | Patients who received ESA treatment within 6 weeks of randomisation were excluded | Roxa: 9.1 g/dL  Pbo: 9.1 g/dL |
| ANDES  (NCT01750190) | <10.0 g/dL | ESA non-users | Patients who received ESA treatment within 12 weeks of study participation were excluded | Roxa: 9.1 g/dL  Pbo: 9.1 g/dL |
| ALPS  (NCT01887600) | The mean of the 3 most recent Hgb values during the screening period, obtained at least 4 days apart: ≤10.0 g/dL, with a difference of ≤1.0 g/dL between the highest and lowest values | ESA non-users | Patients who received ESA treatment within 12 weeks of randomisation were excluded | Roxa: 9.1 g/dL  Pbo: 9.1 g/dL |

(B)

| **Trials** | **Hgb entry range** | **ESA exposure** | **ESA duration** | **Reported baseline Hgb** |
| --- | --- | --- | --- | --- |
| ASCEND-D  (NCT02879305) | 1. 8.0–11.0 g/dL and receiving at least the minimum ESA dose OR  2. >11.0 g/dL to 11.5 g/dL and receiving greater than the minimum ESA dose | ESA exposed  (% N/A) | Use of any approved ESA for at least the 6 weeks prior to screening and between screening and randomisation was required. | Dapro: 10.35 g/dL  RhEpo: 10.39 g/dL |
| ASCEND-TD  (NCT03400033) | 1. 8.0–11.0 g/dL and receiving at least the minimum ESA dose OR  2. >11.0 g/dL to 11.5 g/dL and receiving greater than the minimum ESA dose | 100% ESA exposed | Use of any approved rhEpo or analogue for at least 8 weeks prior to screening and between screening and randomisation was required. | Dapro: 10.44 g/dL  RhEpo: 10.59 g/dL |
| ROCKIES  (NCT02174731) | 1. <12.0 g/dL in patients currently treated with an ESA OR 2. <10.0 g/dL in patients not currently treated with an ESA | Both ESA exposed and limited ESA use | Patients are considered not currently treated if they have not received either epoetin beta pegol (Mircera) for at least 8 weeks or any other ESA for at least 4 weeks prior to visit 1. | Roxa: 9.99 g/dL  RhEpo: 10.02 g/dL |
| SIERRAS  (NCT02273726) | 9.0–12.0 g/dL | 100% ESA exposed | Patients must have been receiving IV or SC ESA for at least 8 weeks prior to screening and on a stable ESA dose during 4 weeks (8 weeks if on epoetin beta pegol [Mircera]) prior to randomisation. | Roxa: 10.25 g/dL  RhEpo: 10.25 g/dL |
| PYRENEES  (NCT02278341) | 9.5–12.0 g/dL | 100% ESA exposed | Patients were on IV or SC epoetin or IV or SC darbepoetin alfa treatment for at least 8 weeks prior to randomisation with stable weekly doses (during 4 weeks prior to randomisation). | Roxa: 10.75 g/dL  RhEpo: 10.78 g/dL |
| INNO2VATE-PD  (NCT02892149) | 1. US: 8.0–11.0 g/dL  2. Non-US: 9.0–12.0 g/dL | ESA exposed  (% NR) | Currently maintained on ESA therapy, with a dose received within 6 weeks prior to or during screening. | Vada: 10.6 g/dL  Darbe: 10.2 g/dL |
| ASCEND-ID  (NCT03029208) | 8.0–11.0 g/dL | Limited ESA use around dialysis initiation | Use of any ESA treatment within 8 weeks prior to screening is prohibited except for limited use as part of dialysis initiation. Limited use is defined as no more than 6 weeks of short acting ESA or long-acting ESA received before or after starting dialysis. | Dapro: 9.46 g/dL  Darbe: 9.49 g/dL |
| HIMALAYAS  (NCT02052310) | ≤10.0 g/dL | ESA naïve (only 6.2% exposed) | Prior ESAs are permitted if received earlier than 12 weeks prior to participating in the study. For US patients, total duration of prior effective ESA use must be less than or equal to 3 weeks within preceding 12 weeks at the time consent is obtained. | Roxa: 8.43 g/dL  RhEpo: 8.46 g/dL |
| INNO2VATE-ID  (NCT02865850) | 8.0–11.0 g/dL | Limited ESA exposure  (% NR) | Limited prior exposure to ESA therapy was permitted. Limited exposure was defined as not having met the criteria for ESA resistance within 8 weeks prior to or during screening. | Vada: 9.37 g/dL  RhEpo: 9.19 g/dL |

Dapro, daprodustat; ESA, erythropoiesis stimulating agents; IV, intravenous; N.A, not applicable; NR, not reported; pbo, placebo; roxa, roxadustat; rhEPO, recombinant human erythropoietin; SC, subcutaneous; US, United States of America; vada, vadadustat.

**Supplementary Table 2.** Clinical trials of daprodustat, roxadustat, and vadadustat used in the network meta-analyses of Hgb change from baseline and risk of MACE in the non-dialysis population with anaemia in CKD.

| **Trial name** | **Interventions** | **Data source** | **Hgb change from baseline** | | | **MACE** | | |
| --- | --- | --- | --- | --- | --- | --- | --- | --- |
|  |  |  | **Outcome and time-period** | **Evaluable population** | **Sample size** | **Outcome and time-period** | **Evaluable population** | **Sample size** |
| ASCEND-ND (NCT02876835) | Daprodustat | CSR data on file; Singh 2021; Post-hoc analysis (ESA non-user subgroup) | Hgb CFB (g/dL) to Week 28–52 | ITT^a^  ESA non-user subgroup of ITT | 1937/1030 | Time to first MACE  (HR and %) | ITT^a^  ESA non-user subgroup of ITT | 1937/1030 |
|  | Darbepoetin |  |  |  | 1935/1032 |  |  | 1935/1032 |
| DOLOMITES (NCT02021318) | Roxadustat | Barratt et al, 2021 | Hgb CFB (g/dL) to Week 28–36 | PPS^b^ | 286 | Time to first MACE  (HR and %) | ITT (on-study) | 323 |
|  | Darbepoetin |  |  |  | 273 |  |  | 293 |
| PRO2TECT CORRECTION (NCT02648347) | Vadadustat | Chertow et al, 2021 | Hgb CFB (g/dL) to Week 24–36 | ITT^c^ | 862 | Time to first MACE  (HR and %) (pooled data from the PRO2TECT trials) | Safety^d^ | 1739 |
|  | Darbepoetin |  |  |  | 863 |  |  |  |
| PRO2TECT CONVERSION (NCT02680574) | Vadadustat | Chertow et al, 2021 | Hgb CFB (g/dL) to Week 24–36 | ITT^c^ | 879 |  |  | 1732 |
|  | Darbepoetin |  |  |  | 872 |  |  |  |

^a^The ASCEND-ND trial defines the ITT population as all the patients who had undergone randomisation. The ASCEND-ND trial assessed Hgb CFB to the average of Weeks 28–52, regardless of adherence to treatment including interruptions and discontinuation, the use of non-randomised ESA medication for any reason including rescue therapy, or the use of blood transfusions; ^b^The DOLOMITES trial defines the PPS population as all patients included in the full analysis set (all randomised patients who received one or more doses of study drug and had one or more post-dose Hgb assessments) who did not meet any PPS exclusion criteria. The complete list of PPS exclusion criteria can be found in the supplementary materials of Barratt et al, 2021. The DOLOMITES trial assessed Hgb CFB to the average of Weeks 28–36 without use of rescue therapy within the 6 weeks prior to and during the 8-week evaluation period; ^c^The PRO2TECT trials define the ITT population as all the patients who had undergone randomisation. The PRO2TECT studies assessed Hgb CFB to the average of Weeks 24–36, regardless of adherence to treatment including interruptions and discontinuation, the use of non-randomised ESA medication for any reason including rescue therapy, or the use of blood transfusions; ^d^The PRO2TECT trials define the safety population as the population of patients randomised and treated. Patients randomised but not treated were excluded.

CFB, change from baseline; CKD, chronic kidney disease; CSR, clinical study report; ESA, erythropoiesis-stimulating agent; Hgb, haemoglobin; HR, hazard ratio; ITT, intention-to-treat; MACE, major adverse cardiovascular event; NCT, National Clinical Trial; PPS, per-protocol set.

**Supplementary Table 3.** Clinical trials of daprodustat, roxadustat, and vadadustat used in the network meta-analysis of SF-36 Vitality in the non-dialysis population with anaemia in CKD.

| **Trial name** | **Interventions** | **Total sample size** | **Data source** | **SF-36 Vitality** | |
| --- | --- | --- | --- | --- | --- |
|  |  |  |  | **Outcome and time-period** | **Evaluable population** |
| ASCEND-NHQ  (NCT03409107) | Daprodustat | 307 | CSR data on file | SF-36 Vitality CFB (score) to Week 28 | ITT^a^ |
|  | Placebo | 307 |  |  |  |
| ALPS  (NCT01887600) | Roxadustat | 389 | Mustafa et al, 2021 | SF-36 Vitality CFB (score) to the average of Week 12 to 28 | FAS^b^ |
|  | Placebo | 203 |  |  |  |
| ANDES  (NCT01750190) | Roxadustat | 608 | Mustafa et al, 2021; Coyne et al, 2021 | SF-36 Vitality CFB (score) to the average of Week 12 to 28 | FAS^b^ |
|  | Placebo | 305 |  |  |  |
| OLYMPUS  (NCT02174627) | Roxadustat | 1384 | Mustafa et al, 2021 | SF-36 Vitality CFB (score) to the average of Week 12 to 28 | ITT^a^ |
|  | Placebo | 1377 |  |  |  |

^a^The ASCEND-NHQ and OLYMPUS trials define the ITT population as all patients who had undergone randomisation; ^b^The FAS consisted of all randomised patients who received at least one dose of study drug and had at least one non-missing post-Hgb assessment.

CFB, change from baseline; CKD, chronic kidney disease; CSR, clinical study report; FAS, full analysis set; Hgb, haemoglobin; ITT, intention-to-treat; NCT, National Clinical Trial; SF-36, 36-Item Short Form Health Survey.

**Supplementary Table 4.** Clinical trials of daprodustat, roxadustat, and vadadustat used in the network meta-analyses of Hgb change from baseline and risk of MACE in the dialysis population with anaemia in CKD.

| **Trial name** | **Interventions** | **Data source** | **Hgb change from baseline** | | | **MACE** | | |
| --- | --- | --- | --- | --- | --- | --- | --- | --- |
|  |  |  | **Outcome and time period** | **Evaluable population** | **Sample size** | **Outcome and time period** | **Evaluable population** | **Sample size** |
| ASCEND-D  (NCT02879305) | Daprodustat | CSR data on file | Hgb CFB (g/dL) to Week 28 to 52 | ITT^a^ | 1487 | Time to first MACE  (HR and %) | ITT^d^ | 1487 |
|  | Darbepoetin |  |  |  | 1477 |  |  | 1477 |
| ASCEND-TD  (NCT03400033) | Daprodustat | CSR data on file | Hgb CFB (g/dL) to Week 28 to 52 | ITT^a^ | 270 | Time to first MACE (%) | ITT^d^ | 270 |
|  | Darbepoetin |  |  |  | 137 |  |  | 137 |
| ASCEND-ID  (NCT03029208) | Daprodustat | CSR data on file | Hgb CFB (g/dL) to Week 28 to 52 | ITT^a^ | 157 | Time to first MACE (%) | ITT^d^ | 157 |
|  | Darbepoetin |  |  |  | 155 |  |  | 155 |
| ROCKIES  (NCT02174731) | Roxadustat | Mustafa et al, 2021 | Hgb CFB (g/dL) to Week 28 to 52 | ITT^b^ | 1051 | Time to first MACE  (HR and %) | ITT^d^ | 1048 |
|  | Epoetin |  |  |  | 1055 |  |  | 1053 |
| SIERRAS  (NCT02273726) | Roxadustat | Mustafa et al, 2021 | Hgb CFB (g/dL) to Week 28 to 52 | ITT^b^ | 370 | Time to first MACE  (HR and %) | ITT^d^ | 370 |
|  | Epoetin |  |  |  | 371 |  |  | 370 |
| PYRENEES  (NCT02278341) | Roxadustat | Mustafa et al, 2021 | Hgb CFB (g/dL) to Week 28 to 52 | ITT^b^ | 413 | Time to first MACE  (HR and %) | OT+7^e^ | 414 |
|  | Darbepoetin/ Epoetin |  |  |  | 420 |  |  | 420 |
| HIMALAYAS  (NCT02052310) | Roxadustat | Mustafa et al, 2021 | Hgb CFB (g/dL) to Week 28 to 52 | ITT^b^ | 522 | Time to first MACE  (HR and %) | ITT^d^ | 522 |
|  | Epoetin |  |  |  | 521 |  |  | 517 |
| INNO2VATE-PD  (NCT02892149) | Vadadustat | Eckardt et al, 2021 | Hgb CFB (g/dL) to Week 40 to 52 | ITT^c^ | 1232 | Time to first MACE  (HR and %) | Safety^f^ | 1777 |
|  | Darbepoetin |  |  |  | 1294 |  |  | 1777 |
| INNO2VATE-ID  (NCT02865850) | Vadadustat | Eckardt et al, 2021 | Hgb CFB (g/dL) to Week 40 to 52 | ITT^c^ | 92 | Time to first MACE  (HR and %) | Safety^f^ | 181 |
|  | Darbepoetin |  |  |  | 100 |  |  | 188 |

^a^The ASCEND trials defined the ITT population as all the patients who had undergone randomisation. The ASCEND trials assessed Hgb CFB to the average of Weeks 28–52, regardless of adherence to treatment; ^b^The ROCKIES, SIERRAS, PYRENEES, and HIMALAYAS trials defined the ITT population as all the patients who had undergone randomisation. The roxadustat trials assessed Hgb CFB to the average of Weeks 28–52 without use of rescue therapy within the 6 weeks prior to and during the 8-week evaluation period; ^c^The INNO2VATE trials define the ITT population as all the patients who had undergone randomisation. The INNO2VATE studies assessed Hgb CFB to the average of Weeks 40–52; ^d^For the ASCEND-D, ASCEND-TD, ASCEND-ID, ROCKIES, SIERRAS, and HIMALAYAS trials, the ITT population was all the patients who had undergone randomisation; ^e^For the PYRENEES trial, the evaluable population was on-treatment plus 7 days; ^f^In the vadadustat trials, the safety population was defined as the population of patients randomised and treated. Patients randomised but not treated were excluded.

CFB, change from baseline; CKD, chronic kidney disease; CSR, clinical study report; Hgb, haemoglobin; HR, hazard ratio; ITT, intention-to-treat; MACE, major adverse cardiovascular event; NCT, National Clinical Trial; OT, on-treatment.

**Supplementary Table 5.** Baseline characteristics in the non-dialysis population with anaemia in CKD.

Please see related Excel file.

**Supplementary Table 6.** Trial-level results for daprodustat, roxadustat, and vadadustat relative to comparator used in the network meta-analyses of efficacy, cardiovascular safety, and quality of life in the non-dialysis population.

| **Efficacy: Hgb change from baseline** | **HIF-PHI** | **LSM difference (95% CI) vs. darbepoetin (g/dL)** |
| --- | --- | --- |
| ***ESA users and ESA non-users at baseline*** | | |
| ASCEND-ND | Daprodustat | 0.10 (0.03, 0.16) |
| DOLOMITES | Roxadustat | 0.02 (-0.13, 0.16) |
| PRO2TECT CORRECTION | Vadadustat | 0.05 (-0.04, 0.15) |
| PRO2TECT CONVERSION | Vadadustat | -0.01 (-0.09, 0.07) |
| ***ESA non-users at baseline*** | | |
| ASCEND-ND (ESA non-users) | Daprodustat | 0.17 (0.09, 0.26) |
| DOLOMITES | Roxadustat | 0.02 (-0.13, 0.16) |
| PRO2TECT CORRECTION | Vadadustat | 0.05 (-0.04, 0.15) |
| **Cardiovascular safety: MACE** | **HIF-PHI** | **HR (95% CI) vs. darbepoetin** |
| ***ESA users and ESA non-users at baseline*** | | |
| ASCEND-ND | Daprodustat | 1.03 (0.89, 1.19) |
| DOLOMITES | Roxadustat | 0.89 (0.60, 1.33) |
| PRO2TECT POOLED | Vadadustat | 1.17 (1.01, 1.36) |
| ***ESA non-users at baseline*** | | |
| ASCEND-ND (ESA non-users) | Daprodustat | 0.95 (0.78, 1.17) |
| DOLOMITES | Roxadustat | 0.89 (0.60, 1.33) |
| **Quality of life: SF-36 Vitality change from baseline** | **HIF-PHI** | **LSM difference (95% CI) vs. placebo (points)** |
| ASCEND-NHQ | Daprodustat | 5.36 (2.17, 8.56) |
| ALPS | Roxadustat | 1.13 (-0.19, 2.44) |
| ANDES | Roxadustat | 1.22 (0.15, 2.29) |
| OLYMPUS | Roxadustat | 0.44 (-0.11, 0.99) |

CI, confidence interval; ESA, erythropoiesis-stimulating agent; Hgb, haemoglobin; HIF-PHI, hypoxia-inducible factor prolyl hydroxylase inhibitor; HR, hazard ratio; LSM, least squares mean; MACE, major adverse cardiovascular event; SF-36, 36-Item Short Form Health Survey.

**Supplementary Table 7.** Baseline characteristics in the dialysis population with anaemia in CKD.

Please see related Excel file.

**Supplementary Table 8.** Trial-level results for daprodustat, roxadustat, and vadadustat relative to comparator used in the network meta-analyses of efficacy and cardiovascular safety in the dialysis population.

| **Efficacy: Hgb change from baseline** | **HIF-PHI** | **LSM difference (95% CI) vs. darbepoetin/epoetin (g/dL)** |
| --- | --- | --- |
| ***Prevalent and incident dialysis*** | | |
| ASCEND-D | Daprodustat | 0.21 (0.15, 0.27) |
| ASCEND-TD | Daprodustat | -0.02 (-0.18, 0.14) |
| ASCEND-ID | Daprodustat | -0.20 (-0.43, 0.04) |
| ROCKIES | Roxadustat | 0.09 (0.01, 0.18) |
| SIERRAS | Roxadustat | 0.48 (0.36, 0.59) |
| PYRENEES | Roxadustat | 0.17 (0.08, 0.26) |
| HIMALAYAS | Roxadustat | 0.18 (0.08, 0.29) |
| INNO2VATE-PD | Vadadustat | -0.18 (-0.25, -0.12) |
| INNO2VATE-ID | Vadadustat | -0.07 (-0.34, 0.19) |
| ***Excluding incident dialysis trials*** | | |
| ASCEND-D | Daprodustat | 0.21 (0.15, 0.27) |
| ASCEND-TD | Daprodustat | -0.02 (-0.18, 0.14) |
| ROCKIES | Roxadustat | 0.09 (0.01, 0.18) |
| SIERRAS | Roxadustat | 0.48 (0.36, 0.59) |
| PYRENEES | Roxadustat | 0.17 (0.08, 0.26) |
| INNO2VATE-PD | Vadadustat | -0.18 (-0.25, -0.12) |
| **Cardiovascular safety: MACE** | **HIF-PHI** | **HR (95% CI) vs. darbepoetin/epoetin** |
| ***Prevalent and incident dialysis*** | | |
| ASCEND-D | Daprodustat | 0.93 (0.81, 1.07) |
| ASCEND-TD | Daprodustat | 1.25 (0.67, 2.33) |
| ASCEND-ID | Daprodustat | 1.25 (0.63, 2.46) |
| ROCKIES | Roxadustat | 1.05 (0.86, 1.29) |
| SIERRAS | Roxadustat | 1.07 (0.77, 1.49) |
| PYRENEES | Roxadustat | 1.43 (0.99, 2.05) |
| HIMALAYAS | Roxadustat | 0.89 (0.62, 1.27) |
| INNO2VATE-PD | Vadadustat | 0.96 (0.83, 1.12) |
| INNO2VATE-ID | Vadadustat | 0.97 (0.54, 1.76) |
| ***Excluding incident dialysis trials*** | | |
| ASCEND-D | Daprodustat | 0.93 (0.81, 1.07) |
| ASCEND-TD | Daprodustat | 1.25 (0.67, 2.33) |
| ROCKIES | Roxadustat | 1.05 (0.86, 1.29) |
| SIERRAS | Roxadustat | 1.07 (0.77, 1.49) |
| PYRENEES | Roxadustat | 1.43 (0.99, 2.05) |
| INNO2VATE-PD | Vadadustat | 0.96 (0.83, 1.12) |

CI, confidence interval; Hgb, haemoglobin; HIF-PHI, hypoxia-inducible factor prolyl hydroxylase inhibitor; HR, hazard ratio; LSM, least squares mean; MACE, major adverse cardiovascular event.
